# Cyclone “Yaku” and *Leptospira* serovars in La Libertad, Peru

**DOI:** 10.17843/rpmesp.2023.404.13261

**Published:** 2023-12-18

**Authors:** Percy Asmat, Manuel Hidalgo, Cynthia Ramos, Pedro Lezama-Asencio, Víctor Fernández-Gómez

**Affiliations:** 1 Human Medicine Study Program, Private University Antenor Orrego, Trujillo, Peru. Private University Antenor Orrego Human Medicine Study Program Private University Antenor Orrego Trujillo Peru; 2 La Libertad Regional Referral Laboratory, Trujillo, Peru. La Libertad Regional Referral Laboratory Trujillo Peru; 3 Department of Science, Private University Antenor Orrego, Trujillo, Peru. Private University Antenor Orrego Department of Science Private University Antenor Orrego Trujillo Peru; 4 Belén Hospital, Trujillo, Peru. Belén Hospital Trujillo Peru

To the Editor. Climate change has impacted Peru in the form of phenomena such as “El Niño”, or the “Yaku” cyclone, which have affected the northern Peruvian coast during 2023. These events have increased temperatures and precipitation, which are related to negative impacts on health ^1^. The high incidence of rainfall caused by these phenomena increases the probability of contact with rodents, the natural reservoirs of *Leptospira* spp., causing contamination of water bodies and soils with these bacteria ^2^. For this reason, this study aimed to characterize *Leptospira* serovars in the La Libertad region, between January and March 2023, in the context of cyclone “Yaku”.

Leptospirosis is one of the most prevalent zoonotic diseases, with high prevalence in tropical areas and a high morbidity rate and thousands of deaths reported annually ^2^. In this scenario, 5123 cases of leptospirosis were reported in Peru during 2022, with 29 cases detected in La Libertad ^3^. The Microscopic Agglutination Test (MAT) was used to detect the cases, which is considered to be the gold standard for the diagnosis of leptospirosis due to the taxonomic complexity of the genus *Leptospira* spp. ^4^.

**Figure 1:**
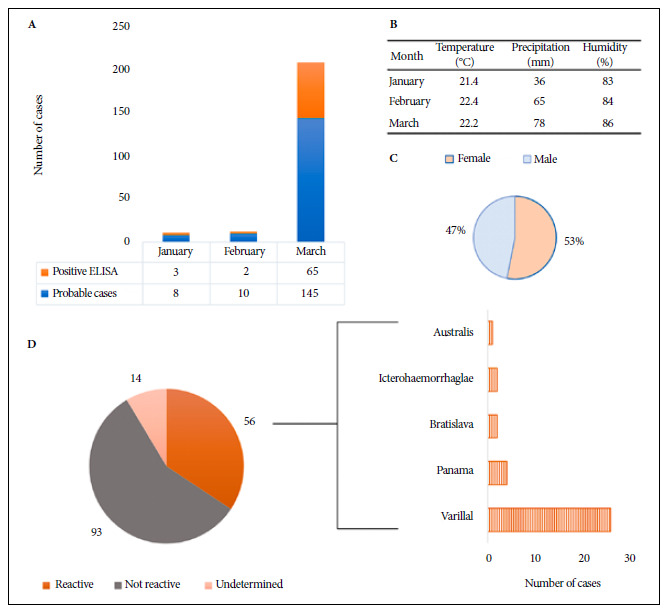
Analysis of *Leptospira* spp. cases at the Regional Referral Laboratory of La Libertad. (A) Probable and positive cases according to ELISA test. (B) Meteorological data on temperature (°C), precipitation (mm) and humidity (%) in La Libertad region. (C) Composition of patients according to sex. (D) MAT results for serovars *Varillal*, *Panama*, *Bratislava*, *Icterohaemorrhagiae* and *Australis*.

Between January and March 2023, during cyclone “Yaku”, 163 samples from patients with presumptive diagnosis of leptospirosis were analyzed at the Regional Referral Laboratory of La Libertad (LRRLL). We report the serological evidence of infection; for this we selected the files with results of the IgM Enzyme-Linked Immunoadsorption Assay (IgM ELISA) reactive for Leptospira. The tests were performed at the LRRLL. Samples with reactive results were screened for MAT evaluation at the National Referral Laboratory for Bacterial Zoonoses of the National Institute of Health in order to determine the serovars with the highest prevalence. The data are presented in Figure 1.

The results show 70 reactive cases screened by ELISA (Figure 1A). Of these, 65 cases were reported in March, coinciding with the highest precipitation rates, according to meteorological data (Figure 1B). Forty-seven percent of the samples were obtained from male patients, while 53% were obtained from females (Figure 1C). The ELISA test yielded positive results for 56 cases and the most prevalent serovars when processed by MAT were *Varillal* (37%), *Panama* (5.7%), *Bratislava* (2.9%), *Icterohaemorrhagiae* (2.9%) and *Australis* (1.4%), as shown in Figure 1D.

The increase of leptospirosis cases during the month of March was probably related to the climatic conditions surrounding cyclone “Yaku”, with high temperatures and increases in environmental humidity, conditions in which, according to Yanagihara *et al*. ^5^, bacteria of the genus *Leptospira* spp. are able to persist indefinitely. Moreover, similar results were reported by Serrano-Martínez *et al*. ^6^, who found a high prevalence rate of *Leptospira* spp. infection under conditions of high humidity and temperature during the spring and summer seasons. In fact, the prevalence of leptospirosis was found to be 28.9% in a district of Chiclayo between October and December 2016 ^7^. Most samples analyzed at LRRLL were associated with the *Varillal* serovar, which coincides with a study conducted at the Public Health Reference Laboratory of Jaén that reported a higher incidence of the *Varillal* and *Icterohaemorrhagiae* serovars under climatic conditions of high rainfall and humidity ^8^. In this context, climatic phenomena such as the cyclone “Yaku” could have serious consequences on public health by providing ideal conditions for the dissemination of infectious agents such as *Leptospira* spp.

The limitations of this study include the fact that MAT was only performed on samples with reactive ELISA results and the presence of only one sample from each patient in the MAT, which limits the study to probable cases according to Health Directive N° 065-MINSA/DGE-V.01. The specific diagnosis of this disease is performed by MAT, polymerase chain reaction (PCR), or bacteriological culture, but these methods are limited by their complexity in order to be implemented. Furthermore, this study does not reflect the magnitude of leptospirosis in the La Libertad region because this was not a prevalence study. Finally, although we found an increase in the number of cases during the study period, specific studies are needed to associate and correlate the magnitude of the influence of climate change with the appearance of leptospirosis.

In conclusion, the number of leptospirosis cases increased when cyclone “Yaku” appeared, mostly during March, which is when the cyclone impacted La Libertad region. In this context, it is important to note that most *Leptospira* infection cases were due to the *Varillal* and *Panama* serovars, therefore we consider that it is necessary to adopt promotion and prevention measures, avoiding their spread and contributing to improve public health in Peru. These measures should include the development of rapid immunochromatographic tests of high sensitivity and specificity that can be applied at the first level of care, especially under adverse weather conditions.
