# Supplementary material for: Cyclone “Yaku” and Leptospira serovars in La Libertad, Peru
Source: Rev Peru Med Exp Salud Publica. 2023 Dec 18;40(4):495–7. doi: 10.17843/rpmesp.2023.404.13261 (PMC11138826; doi:10.17843/rpmesp.2023.404.13261)
Supplement: Translation

# Translation: El ciclón «Yaku» y serovares de *Leptospira* en La Libertad, Perú

**Authors:** Percy Asmat, Manuel Hidalgo, Cynthia Ramos, Pedro Lezama-Asencio, Víctor Fernández-Gómez

**Affiliations:** 1 Programa de Estudio de Medicina Humana, Universidad Privada Antenor Orrego, Trujillo, Perú.; 2 Laboratorio de Referencia Regional La Libertad, Trujillo, Perú.; 3 Departamento de Ciencias, Universidad Privada Antenor Orrego, Trujillo, Perú.; 4 Hospital Belén, Trujillo, Perú.

Sr. Editor. El cambio climático se ha manifestado en Perú en forma de fenómenos como «El Niño», o el ciclón «Yaku», que han afectado a la costa norte de este país durante el año 2023. Estos eventos han incrementado las temperaturas y precipitaciones, relacionándose con impactos negativos en la salud ^(^^1^. La mayor incidencia de lluvias generada por estos fenómenos incrementa la probabilidad de contacto con roedores, los reservorios naturales de *Leptospira* spp., ocasionando contaminación de cuerpos de agua y suelos con estas bacterias ^2^. Por esta razón, el objetivo de este trabajo es realizar la caracterización de serovares de *Leptospira* en la región La Libertad, entre enero y marzo de 2023, en el contexto del ciclón «Yaku».

La leptospirosis es una de las enfermedades zoonóticas más prevalentes, con una alta tasa de morbilidad y miles de muertes reportadas anualmente, con mayor prevalencia en zonas tropicales ^2^. En este escenario, durante el año 2022, en Perú se reportaron 5123 casos de leptospirosis, con 29 casos detectados para La Libertad ^3^. Esta detección se realiza mediante el Test de Aglutinación Microscópica (MAT, por sus siglas en inglés), que por la complejidad taxonómica del género *Leptospira* spp., y es considerado el estándar de oro para el diagnóstico de leptospirosis ^(^^4^.

En el Laboratorio de Referencia Regional de La Libertad (LRRLL), se analizaron 163 muestras de pacientes con diagnóstico presuntivo de leptospirosis en el marco de aparición del ciclón «Yaku», entre enero y marzo de 2023. La evidencia serológica de infección se reporta en este trabajo, para lo cual, se seleccionaron las fichas que mostraban resultados del Ensayo por Inmunoadsorción Ligado a Enzimas de IgM (ELISA IgM) reactivas para *Leptospira*, prueba realizada en el LRRLL. Las muestras con resultados reactivos fueron tamizadas para su evaluación MAT en el Laboratorio de Referencia Nacional de Zoonosis Bacterianas del Instituto Nacional de Salud para determinar los serovares con mayor prevalencia. Los datos se presentan en la Figura 1.

Los resultados muestran 70 casos reactivos tamizados por ELISA (Figura 1A). Del total, 65 casos fueron reportados en marzo, coincidiendo con las tasas de precipitación más elevadas, según los datos meteorológicos (Figura 1B). El 47% de las muestras provinieron de pacientes varones, en tanto que el 53% provino de mujeres (Figura 1C). La prueba ELISA, arrojó resultados positivos para 56 casos, los cuales al ser procesados por la prueba MAT muestran que los serovares más prevalentes fueron *Varillal* (37%), *Panama* (5,7%), *Bratislava* (2,9%), *Icterohaemorrhagiae* (2,9%) y *Australis* (1,4%), tal como se muestra en la Figura 1D.

**Figura 1:** Análisis de casos de *Leptospira* spp. en el Laboratorio de Referencia Regional de La Libertad. (A) Casos probables y positivos según la prueba ELISA. (B) Datos meteorológicos de temperatura (°C), precipitación (mm) y humedad (%) en la región La Libertad. (C) Composición de los pacientes según sexo. (D) Resultados de MAT con los serovares *Varillal, Panama, Bratislava, Icterohaemorrhagiae* y *Australis*

El incremento de casos de leptospirosis observado durante el mes de marzo podría estar relacionado con las condiciones climáticas en torno al ciclón «Yaku», con mayores temperaturas acompañadas de incrementos en la humedad ambiental, condiciones en las cuales, según Yanagihara *et al.*^5^, las bacterias del género *Leptospira* spp. son capaces de subsistir de manera indefinida. Además, resultados similares fueron reportados por Serrano-Martínez *et al.*^6^ con mayor presencia de infecciones por *Leptospira* spp. en condiciones de alta humedad y temperatura correspondientes a las estaciones de primavera y verano. De hecho, en un distrito de Chiclayo, entre octubre y diciembre de 2016, se encontró una prevalencia del 28,9% de leptospirosis ^7^. En el caso de las muestras analizadas en el LRRLL, la mayoría de los casos estuvo asociada con el serovar *Varillal*, lo cual coincide con un estudio realizado en el Laboratorio Referencial de Salud Pública de Jaén, donde se encontró una mayor incidencia de los serovares *Varillal* e *Icterohaemorrhagiae* en condiciones climáticas de alta precipitación y humedad ^8^. En este contexto, los fenómenos climáticos como el ciclón «Yaku» podrían tener consecuencias graves sobre la salud pública al brindar las condiciones para la multiplicación de agentes infecciosos como *Leptospira* spp.

Las limitaciones incluyen la realización del MAT solo en las muestras con resultados de ELISA reactivos, la presencia de solo una muestra de cada paciente en la prueba MAT, lo que limita el estudio a casos probables según la Directiva Sanitaria N° 065-MINSA/DGE-V.01. También se debe tener en cuenta que el diagnóstico específico de esta enfermedad se realiza mediante MAT, la reacción en cadena de la polimerasa (PCR, por sus siglas en inglés) o el cultivo bacteriológico, para los cuales existen limitaciones por la complejidad de su implementación. Además, este trabajo no refleja la magnitud de la leptospirosis en la región La Libertad al no ser un estudio de prevalencia. Por último, si bien, durante el periodo de estudio se observó un incremento de casos, se requieren de estudios específicos que asocien y correlacionen la magnitud de la influencia del cambio climático con la aparición de leptospirosis.

En conclusión, se reporta un alto número de casos de leptospirosis en el marco de aparición del ciclón «Yaku», destacando el incremento observado en marzo, mes en el que la región La Libertad fue afectada por este fenómeno climático. En este contexto, destaca la presencia de los serovares *Varillal* y *Panama* de *Leptospira* con mayor número de casos, por lo cual el equipo de trabajo considera que es necesario adoptar medidas de promoción y prevención, evitando su propagación y contribuyendo a mejorar la salud pública en Perú. Dentro de estas medidas, se puede considerar el desarrollo de pruebas inmunocromatográficas rápidas de alta sensibilidad y especificidad que puedan aplicarse en el primer nivel de atención, sobre todo en condiciones climáticas adversas.
